# Deep tissue volume imaging of birefringence through fibre-optic needle probes for the delineation of breast tumour

**DOI:** 10.1038/srep28771

**Published:** 2016-07-01

**Authors:** Martin Villiger, Dirk Lorenser, Robert A. McLaughlin, Bryden C. Quirk, Rodney W. Kirk, Brett E. Bouma, David D. Sampson

**Affiliations:** 1 Harvard Medical School and Massachusetts General Hospital, Wellman Center for Photomedicine, Boston, MA USA; 2 Optical+Biomedical Engineering Laboratory, The University of Western Australia, Perth, WA 6009, Australia; 3 Harvard-Massachusetts Institute of Technology, Program in Health Sciences and Technology, Cambridge, MA 02142, USA; 4 Centre for Microscopy, Characterisation and Analysis, The University of Western Australia, Perth, WA 6009, Australia

## Abstract

Identifying tumour margins during breast-conserving surgeries is a persistent challenge. We have previously developed miniature needle probes that could enable intraoperative volume imaging with optical coherence tomography. In many situations, however, scattering contrast alone is insufficient to clearly identify and delineate malignant regions. Additional polarization-sensitive measurements provide the means to assess birefringence, which is elevated in oriented collagen fibres and may offer an intrinsic biomarker to differentiate tumour from benign tissue. Here, we performed polarization-sensitive optical coherence tomography through miniature imaging needles and developed an algorithm to efficiently reconstruct images of the depth-resolved tissue birefringence free of artefacts. First *ex vivo* imaging of breast tumour samples revealed excellent contrast between lowly birefringent malignant regions, and stromal tissue, which is rich in oriented collagen and exhibits higher birefringence, as confirmed with co-located histology. The ability to clearly differentiate between tumour and uninvolved stroma based on intrinsic contrast could prove decisive for the intraoperative assessment of tumour margins.

Breast cancer is, by far, the most frequent cancer among women, with an incidence rate greater than 70 in 100,000 women per year in the developed world^1^. Population screening programs have helped to improve the survival rate but breast cancer remains the second leading cause of cancer-related death in women in the developed world. Surgical removal of malignant tissue offers good prognosis when malignancy is detected at a sufficiently early stage. However, a persistent challenge in such breast-conserving surgery is the delineation of tumour margins, which is necessary to ensure that the entire tumour is resected. Margin involvement during surgery remains problematic^2^ and the gold standard, assessment by histology, does not provide feedback during the operation. Involved margins or inadequate clearance occur in around 25–30% of patients (up to 60% has been reported) and result in an increased risk of local recurrence or require further surgery^3^,^4^. Additional surgeries generate considerable psychological, physical and economic stress, and can delay recommended adjuvant therapies^5^.

Working towards assessing these margins in an intraoperative setting and guiding the resection, we have previously developed fibre-optic imaging probes for optical coherence tomography (OCT), and integrated them into thin hypodermic needles^6^,^7^,^8^. OCT provides cross-sectional views of the subsurface microstructure of biological tissue, at high spatial resolution of <15 μm in the axial and lateral directions. Spatial variation in the sample’s refractive index causes scattering of the incident light and generates intrinsic contrast without the need for any labelling. Scattering, however, also limits the imaging depth to 1–2 mm. Integration into needle probes permits imaging at greater depths below the tissue surface, and has enabled structural imaging deep inside tissue, including in breast tumour samples^9^. In breast tissue, it is straightforward to identify adipose tissue due to its characteristic structural signal^10^. However, scattering contrast between malignant tissue and the surrounding, uninvolved connective tissue is limited and renders the clear identification and delineation of the tumour challenging^9^. Connective tissue and the dense, fibrous desmoplastic stroma frequently surrounding malignant regions primarily consist of collagen. Such fibrillar collagen exhibits birefringence, an optical property that results in a differential delay, or retardation, between electromagnetic waves propagating with their transverse components polarized along or orthogonal to the collagen fibres.

Polarization sensitive (PS)-OCT has long been used to measure the polarization states of the light backscattered by tissue^11^,^12^. Observing how the measured polarization states vary as a function of depth, one can obtain a measure of tissue birefringence. This has been used extensively in ophthalmology^13^, for imaging of skin burns^14^,^15^ and scars^16^, and in birefringent structures such as tendons^17^ and myelinated nerve fibres^18^. It has also shown promise for the assessment of atherosclerotic lesions^19^, muscle fibre integrity^20^, and orientation^21^. In order to unambiguously characterize the polarization response of the tissue and also enable fibre-based imaging, the illumination has to be varied between at least two distinct input polarization states^22^. Recently, a simple and efficient hardware implementation, that uses only passive components to encode two input polarization states along depth, has been introduced^23^,^24^,^25^. Currently, the majority of PS-OCT studies employ the Jones formalism, and compute the (cumulative) retardation from the sample surface to a given depth. This results in retardation images that are difficult to interpret and convey little insight when the orientation of the optic axis varies in depth^26^. Reconstructing instead the depth-resolved tissue birefringence by computing the local retardation between axially adjacent pixels offers a more intuitive view of the tissue features that give rise to increased retardation^27^,^28^. This strategy is more susceptible to measurement noise, as it is related to taking the derivative of the cumulative polarization matrix. Accordingly, averaging would be beneficial to improve the accuracy of the measured polarization states, but this has proven to be challenging to perform in the coherent Jones formalism^29^,^30^,^31^,^32^. Also, each sample layer is imaged through all the preceding tissue, and it is, in general, only possible to retrieve a similarity transformation of the polarization matrix of a sample layer, i.e., a matrix that in linear algebra is called ‘similar’ to that of the given sample layer^28^. This results in accurate local retardation values only when all tissue layers act as pure retarders, which is not necessarily the case^27^,^33^.

In this work, we combined, for the first time, PS-OCT with imaging through miniature needle probes. We built a portable, passively depth-encoded PS-OCT system to interface with our imaging needles, and developed a novel reconstruction method based on the Mueller formalism: Mueller matrices were constructed from their corresponding measured Jones matrices and incoherently averaged before extracting the local retardation using the differential Mueller formalism^34^,^35^. This approach overcomes difficulties with averaging in the coherent Jones formalism and results in improved and robust measurements of depth-resolved tissue birefringence. We also describe a metric to assess the accuracy of the similarity transformation beyond the assumption of tissue being a pure retarder. Imaging fresh, excised tumour specimens with needle-based PS-OCT, we found compelling contrast between more highly birefringent uninvolved stroma and lowly birefringent malignant regions, which we confirmed with spatially co-registered histology. These results demonstrate the potential of using intrinsic tissue birefringence as a biomarker to differentiate between healthy tissue and tumour, which could aid in the intraoperative assessment of breast tumour margins.

## Results

### Needle-based PS-OCT system

**Figure 1:**
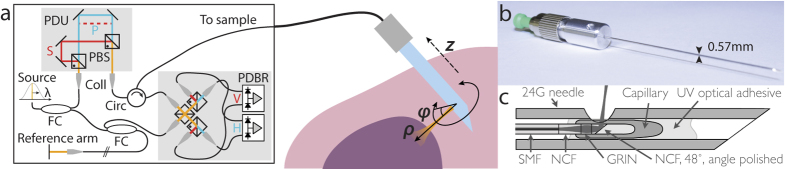
(**a**) Schematic of the PS-OCT system and the principle of needle-based imaging. The wavelength-swept laser source was connected to a fibre-based interferometer, containing a polarization delay unit (PDU) to encode the two orthogonal input polarization states S and P at distinct path length differences. In combination with the polarization-diverse balanced receiver (PDBR), the full polarization response of the sample was retrieved. The imaging system was interfaced to a side-viewing needle probe, which scans a cylindrical tissue volume through a combined rotation and pullback motion. PBS: Polarizing beam splitter; Coll: Collimator; Circ: Circulator; FC: Fibre coupler. (**b**) Photograph and (**c**) schematic of the employed 24G imaging needle. SMF: Single mode fibre; NCF: No-core fibre; GRIN: Graded index fibre.

In order to combine our previously developed imaging needles^8^ with polarization-sensitive measurements, we modified an existing 1310-nm swept-source OCT system by implementing a polarization-diverse receiver and passively multiplexing in depth two orthogonal illumination polarization states in the sample arm (Fig. 1), similar to refs 23, 24, 25. A related approach has been previously coined ‘coherence multiplexing’ and used in telecommunications and optical fibre sensors^36^. Unlike active modulation of the polarization states, which requires additional instrumentation and complex synchronization, passive multiplexing offers a simple and effective strategy to probe the full polarization response of the sample within a single A-line. However, this approach relies on an adequate imaging range to accommodate the depth-encoded signals. This range is set by the sufficiently narrow instantaneous line width of the employed wavelength-swept light source (AXP50125-6, Axsun, Billerica, USA) that offered a 6 dB intensity roll-off of almost 8 mm. Since the available imaging range is proportional to the inverse of the acquisition rate at which the wavelength-swept signal is sampled, we electronically frequency-doubled the original sampling clock provided by the Axsun light source for optical clocking of the acquisition, thereby doubling the default imaging range from 5 mm to 10 mm. Timing jitter between adjacent A-lines leads to a relative phase offset between the multiplexed input polarization states. This offset was detected and removed and the remaining depth-dependent signal roll-off was corrected from a calibration measurement (see Methods for details).

The 24-gauge (24G, 570 μm outer diameter) imaging needles used in this study were connected to the sample-arm fibre of the PS-OCT system via an FC/APC fibre connector and mounted on motorized actuators that scanned the sample with a combined rotation and pullback of the needles. This resulted in the sampling of the tissue volume in cylindrical coordinates, reporting the backscatter signal as function of angular position φ and radial distance *ρ* from the needle, along the pullback direction *z*, as illustrated in Fig. 1. The resolution of the OCT system was 13 μm in tissue in the axial, and ~12 μm in the lateral directions, assuming a refractive index *n* = 1.36 of the tissue. Remapping into Cartesian coordinates then revealed the undistorted sample geometry.

### Reconstruction of sample birefringence

The reconstructed complex-valued tomograms of the individual channels of the PS-OCT system provide a measure of the round-trip Jones matrix from the polarization delay unit to a given sample depth and on to the receiver. This Jones matrix contains the cumulative polarization information on propagation through the individual components of the imaging system and layers of the tissue. At each pixel, the accuracy of the measured polarization states depends on the signal-to-noise ratio (SNR). With the high prevalence of low signal pixels in speckle-dominated tomograms typical of OCT, averaging between adjacent pixels is needed to improve the accuracy of the measured polarization states^37^. Spatial averaging in the Jones formalism, which describes coherent and fully polarized light fields, requires special attention to adjust the global phase between the Jones matrices of adjacent pixels to avoid unwanted interference effects^25^,^30^,^31^,^38^. In contrast, the Mueller formalism describes intensity-based quantities and encompasses incoherent and partially polarized light. Spatial averaging is straightforward in the Mueller formalism, and equivalent to suppressing speckle by incoherent averaging. We, thus, transformed the Jones matrix measured at each pixel into its corresponding Mueller-Jones matrix. This Mueller-Jones matrix is equivalent to the original Jones matrix in that it contains the same retardation and diattenuation information. Importantly, the global phase value that creates interference effects in the Jones formalism is removed. Each element of the calculated Mueller matrices was then spatially filtered with a Gaussian kernel, extending roughly two speckle widths in the axial and lateral direction, to obtain **M**(*z*). Unlike the originally constructed Mueller-Jones matrix, this filtered Mueller matrix no longer has a direct correspondence in the Jones formalism and belongs to a more general class of Mueller matrices^39^,^40^. In addition to retardation and diattenuation, it also describes depolarization, which can provide additional contrast^41^.

In analogy with PS-OCT in the Jones formalism^28^, to obtain a measure of the depth-resolved tissue birefringence, we aimed to compute the product of the Mueller matrix and its inverse at a differential depth ∆**M**(*z*) = **M**(*z* + ∆*z*/2)⋅**M**^−1^(*z* − ∆*z*/2). To accelerate the reconstruction and to avoid the computationally demanding matrix inversion, we constructed a pseudo-inverse, which corresponds to the true inverse for a Mueller-Jones matrix, but only an approximation thereof for a general Mueller matrix (see Methods for details). We then used the differential Mueller matrix formalism^34^,^35^ to extract the retardation *γ* from ∆**M**(*z*). Unlike the frequently employed polar decomposition^42^, for which a specific but ambiguous ordering of retarding, diattenuating, and depolarizing elements has to be chosen to decompose ∆**M**(*z*), the differential Mueller formalism assumes the concurrent action of these effects and removes this ambiguity. The retardation *γ* was then converted to tissue birefringence ∆*n*, denoting the difference of the refractive index along the fast and slow axis of propagation, by ∆*n = γ*/(*k*_*c*_2∆z), where *k*_*c*_ is the mean wavenumber in vacuum, ∆z the differential depth over which the retardation was evaluated, and the factor two takes into account the double pass through the sample.

**Figure 2:**
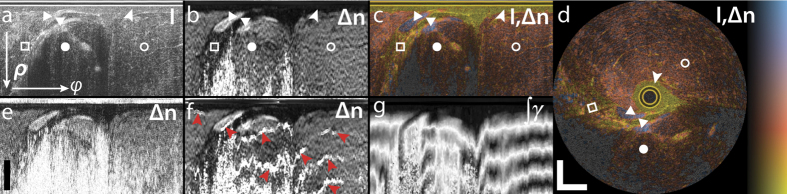
Reconstructed sample birefringence from PS-OCT measurements of a fish specimen. (**a**) Logarithm of structural intensity signal of one rotational scan, scaled from −10 to 40 dB of SNR (I). (**b**) Depth-resolved tissue birefringence (Δn) obtained with Mueller-based processing. (**c**) Overlay of structural intensity signal and tissue birefringence (I,Δn) using isoluminant colourmap, displaying the birefringence as colour hue and the intensity as brightness. In regions with a depolarization index smaller than 0.3, only the greyscale intensity data is displayed. (**d**) Mapping the same cross-section from polar to Cartesian coordinates reveals the undistorted tissue architecture. The birefringence clearly delineates a first layer of liquid (arrowheads shown in **a**–**d**), which is non-birefringent, from the muscle tissue, which exhibits birefringence of ~1.8 × 10^−3^. Two fish-bones stand out with a more pronounced birefringence (triangles), below which appears a region of fatty tissue with decreased birefringence (white squares). Muscle tissue features a uniform birefringence, also in regions of lower intensity signal (full circles), as compared to locations of higher intensity signal (empty circles). (**e**) Local tissue birefringence without spatial averaging suffers from limited SNR and does not recover the birefringence in deeper lying tissue regions. (**f**) Local tissue birefringence obtained with coherent averaging of Jones matrices results in artefacts (red arrowheads). (**g**) Cumulative tissue birefringence (∫*γ*) is difficult to interpret and conceals the layered sample architecture. Scale bars are 500 μm, assuming a refractive index of tissue of *n* = 1.36. Colour range indicates birefringence of 0.18 × 10^−3^–2.2 × 10^−3^, brightness −10–40 dB.

Figure 2 shows the structural intensity signal and the tissue birefringence imaged by inserting an imaging needle into the flank of a fish (Black Bream, Acanthopagrus butcheri). The fish was acquired fresh from a local supplier and kept refrigerated (not frozen) before imaging. The structural intensity signal is obtained by taking the determinant of the Jones matrix at each pixel^29^. To combine the structural and birefringence signal in a single view, we use an isoluminant colourmap, where the birefringence is mapped to a colour hue and the structural signal to brightness^43^ (Fig. 2c,d). The birefringence helps to identify individual sample layers and tissue structures, and provides contrast complementary to the structural intensity signal. From **M**(*z*), we also compute the depolarization index^41^ at each sample location. Regions with low SNR are dominated by noise and cause a low depolarization index and random birefringence measurements. To mask such areas, we displayed only the greyscale intensity signal for regions with a depolarization index <0.3.

Figure 2b visualizes the benefit of spatial averaging by comparison with the birefringence recovered from the original Mueller-Jones matrices without any averaging, shown in Fig. 2e. Although similar birefringent structures are visible, averaging dramatically improves the contrast between tissue with lower and higher birefringence, reduces noise, and more reliably recovers birefringence from larger depths. With the current averaging kernel, these improvements cost a ~two-fold reduction in the spatial resolution along both the axial and lateral directions. Figure 2f indicates the artefacts that arise from coherently averaging Jones matrices, even when adjusting the phase offset, following here the strategy proposed by Li *et al*.^32^. Although this birefringence image corresponds in most regions closely to the one recovered using the Mueller formalism, there are a few locations where the sign of the corrected Jones matrices abruptly changes, which causes the observed bands of overestimated birefringence. Frequently, PS-OCT data is analysed by reconstructing the cumulative retardation from the sample surface to a given sample depth. Because of the high SNR at the sample surface, this reconstruction generally is more robust than the reconstruction of depth-resolved retardation. However, in tissue with preceding birefringent layers with distinct optic axis orientations, the cumulative retardation produces a very convolved picture of the sample birefringence. As shown in Fig. 2g, the cumulative retardation is difficult to interpret and conceals the fine details clearly visible in the depth-resolved birefringence image.

**Figure 3:**
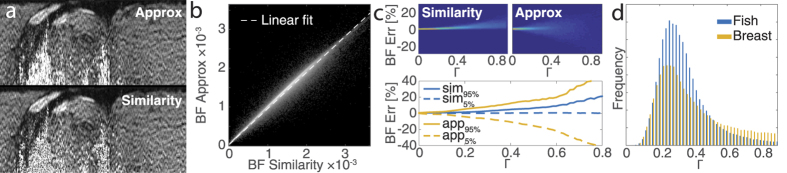
(**a**) Comparison of tissue birefringence retrieved with the approximated (Approx) and the accurate (Similarity) similarity transformation. (**b**) Logarithm of the frequency of co-occurrence of the birefringence values obtained with the approximated and the accurate similarity transformation. In sample regions with a depolarization index ≥0.3, these birefringence values correlate with an r^2^ = 0.995, and a slope of 0.934, showing excellent agreement. (**c**) Simulations of the expected error of the reconstructed birefringence (BF Err) under the accurate and approximated similarity transformation as a function of Γ^2^ = κ^2^ + 3/2(d_1_^2^ + d_2_^2^) (see text for definition of terms). The bottom plot indicates the 5% and 95% confidence levels of the error distribution as a function of Γ. (**d**) Experimental Γ values for fish and breast tumour specimens in regions with a depolarization index ≥0.3 indicate that the approximated similarity transformation results in errors <10% in the majority of both samples.

To verify the validity of the approximated matrix inversion, we compare in Fig. 3 the reconstructed birefringence values to those obtained from the exact, slow reconstruction that uses the accurate matrix inverse. The values correlate with an r^2^ = 0.995, when fitting a linear slope with robust linear least squares fitting (slope of 0.934 and offset of 2.75 × 10^−5^). This demonstrates excellent agreement between the two computations, suggesting that the approximation is sufficiently good, at least for the given sample. In this analysis, in analogy with displaying the birefringence colourmap, pixels with a depolarization index <0.3 were excluded to mask regions that are dominated by noise.

In addition to the inaccuracy due to the approximation of the inverse, it is important to investigate the limitations of using the similarity transformation to extract the local retardation. The retrieved differential Mueller matrix ∆**M** = **Q**⋅∆**M′**⋅**Q**^−1^ corresponds to the similarity transformation of the true local sample matrix ∆**M′**, with **Q** the single pass matrix describing propagation from a given sample depth to the receiver (see Methods for details), i.e., each sample layer is imaged through all the preceding tissue. The similarity transform preserves eigenvalues, but not eigenvectors, and the implications of this have been discussed in the Jones formalism^28^. It has even been shown that it is possible to isolate ∆**M′** by compensating for **Q** in a complicated iterative procedure^21^,^44^, but this approach is incompatible with current fibre-based PS-OCT systems, where the propagation through the fibre to and from the sample alters the polarization states. However, if **Q** is a pure retarder and accordingly a unitary transformation, its only effect will be to change the orientation of the apparent optic axis, without altering the extracted amount of scalar birefringence^28^. On the other hand, if **Q** also contains diattenuation and/or depolarization, then the recovered birefringence is affected. To analyse and quantify this effect, we decomposed the round trip matrix **Q**⋅**D**⋅**Q**^T^⋅**D** = **L**_2_⋅**K**⋅**L**_1_ into its canonical form, where **L**_1,2_ are orthochronous Lorentz transforms (Mueller-Jones matrices with only retardation and diattenuation and det(**L**_1,2_) = 1), and **K** = diag(*K*_0_, *K*_0_*K*_1_, *K*_0_*K*_2_, *K*_0_*K*_3_) is a diagonal matrix, where we limited 0 ≤ *K*_1,2,3_ ≤ 1^40^,^45^. **D**⋅**Q**^T^⋅**D** defines the reversed propagation through **Q**, passing from the sample surface to the given sample depth, where **D** = diag(1, 1, 1, −1). This is equivalent to taking the matrix transpose in the Jones formalism. Simulating the error of the recovered birefringence as a function of **L**_1,2_ and **K** confirmed that the retardation of **L**_1,2_, as well as the overall attenuation *K*_0_, have no influence. Also, isotropic depolarization, for which *K*_1_ = *K*_2_* = K*_3_, is correctly compensated for by our pseudo-inverse of ∆**M**. However, increasing diattenuation of **L**_1,2_ as well as anisotropic depolarization of *K*_1,2,3_ results in a growing error of the recovered retardation. Defining a parameter *Γ*^ 2^ = *κ*^2^ + 3/2(*d*_1_^2^ + *d*_2_^2^), where *κ* is the angle between the vector [*K*_1_, *K*_2_*, K*_3_]^T^ and the isotropic vector [1, 1, 1]^T^, and *d*_1,2_ are the diattenuations of **L**_1,2_, respectively, we can visualize the error between the retardation of the measured ∆**M** and the true ∆**M′**, as shown in Fig. 3c. These error distribution functions were obtained by generating 5 × 10^5^ realizations of randomly generated **Q**-matrices and computing the corresponding similarity transformation and its approximated version of a randomly generated linear retarder ∆**M′**.

The accurate similarity transformation without approximation of the inverse constantly overestimates the retardation. With an increasing *Γ*, both the bias and the spread of the possible errors grow. When using the approximated inverse, the error is less biased, but the spread increases more aggressively with *Γ*. However, even in this case, the error only exceeds 20% of the nominal birefringence for *Γ *≥* *0.6. Using experimental data of fish tissue and breast tumour samples, we decomposed the averaged general Mueller matrices into their canonical forms to compute the parameter *Γ*. As visualized in Fig. 3d, more than 80% of the sample Mueller matrices with a depolarization index ≥0.3 feature a *Γ* ≤ 0.6. With the majority of the sample featuring values around 0.25, the resulting errors for these sample locations are bound to less than 10%. Regions with very low SNR can result in a large *Γ*, but at the same time correspond to low depolarization values and are identified in our analysis with a threshold of 0.3 on the depolarization index. In summary, although some amount of diattenuation and depolarization is present in the experimental measurements of breast tissue, their values are sufficiently low for the method of evaluation of depth-resolved birefringence to yield accurate results.

### Imaging of breast tumour specimens

**Figure 4:**
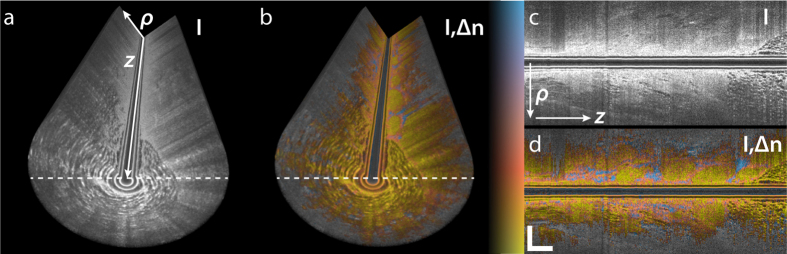
Birefringence imaging of excised breast tumour sample exhibiting patches of low birefringence that are surrounded by regions of higher birefringence. (**a**) Volume rendering of structural intensity signal (I) and (**b**) overlay of birefringence and structural signal (I,Δn). (**c**) Longitudinal cross-section (radial distance from needle versus pullback) of structural intensity signal (I) and (**d**) corresponding overlay (I,Δn), indicated in (**a,b**) by dashed lines. Scale bars are 500 μm, assuming a refractive index of tissue of *n* = 1.36. Colour range indicates birefringence of 0.18 × 10^−3^–2.2 × 10^−3^ and regions with a depolarization index <0.3 are displayed in greyscale only. Brightness range is −10–40 dB.

Next, we moved the mobile, needle-based PS-OCT system to Sir Charles Gairdner Hospital in Perth, Western Australia, for a pilot imaging study. We imaged two freshly excised tumour specimens of invasive ductal carcinomas, retrieved from a lumpectomy and a mastectomy, respectively. The specimens were scheduled to undergo routine histopathology to assess margin involvement, and were available for needle-based imaging prior to sample fixation. We inserted the needle into the suspected centre of the tumour and performed 5 mm-long pullbacks while counter-rotating the needle. Before retrieving the imaging needle, a second needle was inserted adjacent and in parallel to the imaging needle and remained in the tissue during fixation in formalin to guide sectioning of the histology. Figure 4 presents a typical reconstructed data set. In the intensity image, it is simple to identify adipose tissue, with its characteristic features of signal void regions surrounded by highly scattering interfaces, corresponding to lipid-filled adipocytes. On the other hand, the uninvolved connective and tumour tissues generated a homogenous scattering signal. Although there are some regions with distinct grey levels, it is challenging to identify individual structures. The tissue birefringence, in contrast, reveals a clear patterning within this region of uniform structural signal intensity, defining patches of low birefringence that are surrounded by regions of higher birefringence. With the birefringence image available, it is then possible to associate some, but not all, of the lowly birefringent regions with a slightly reduced scattering signal. At larger depths, the propagation of the probing light through the tissue and the rapidly declining SNR result in sufficient depolarization to make the recovery of tissue birefringence unreliable. Regions with a depolarization index below 0.3 are displayed in grey scale only to mask these areas.

**Figure 5:**
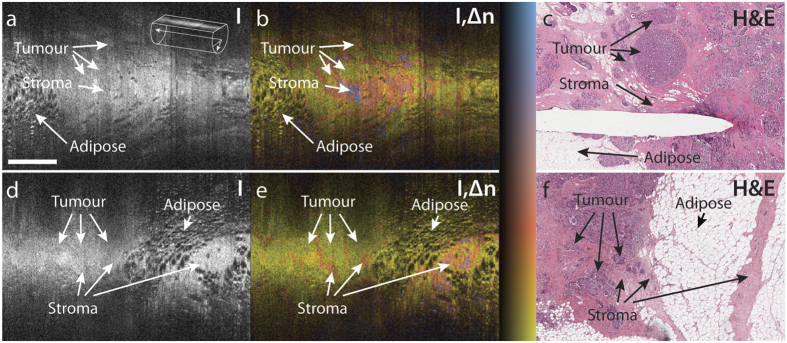
Comparison of oblique cross-sections with matching histology of *ex-vivo* breast tumour samples. (**a,d**) Structural intensity (I), (**b,e**) overlay of tissue birefringence and intensity (I,Δn), and (**c,f**) matching histological section stained with haematoxylin and eosin (H&E). (**a–c**) are from a wide local excision (lumpectomy) of a 20 mm, grade 1 invasive ductal carcinoma. (**d–f**) are from a mastectomy of a 20 mm, grade 2 invasive ductal carcinoma. The needle track visible in (**c**) is due to a needle inserted into the tissue after imaging to guide the collection of histology, and hence does not appear in the OCT images. Scale bar is 1 mm and applies to all panels. Colour range indicates birefringence of 0.18 × 10^−3^–2.2 × 10^−3^. Brightness range is −10–40 dB.

To directly compare the observed birefringence features to histology, we retrieved oblique sections from the imaged tissue cylinders that match the plane of the histological sections, as displayed in Fig. 5. This comparison enabled the clear identification of zones of low birefringence and uniform structural signal as areas of malignant tissue, and the association of the higher birefringence signal with non-cancerous, desmoplastic or stromal regions. This clear match with histology confirms our assumption, that birefringence can serve as an intrinsic biomarker to differentiate malignant from surrounding connective tissue.

## Discussion

Breast tissue contains lobules of alveolar glands and ducts that are supported by a dense fibrous connective tissue, termed fibrous stroma, and lipid-filled adipocytes (fat) in varying proportions. Breast cancer arises in either the ducts or the lobules, and eventually invades the surrounding tissues. In addition, tumours frequently trigger a desmoplastic response, which leads to additional growth of dense fibrous tissue surrounding the tumour. Hence, malignant lesions are surrounded by a combination of fibrous or desmoplastic stroma and fat. To ensure the complete removal of tumour during surgery, the resected tissue is processed for histology and analysed by a pathologist for adequate clearance. Formalin fixation is preferred, because frozen sections give poor results on samples with substantial amounts of adipose tissue. This process takes up to a week before the surgeon receives feedback and can schedule a re-excision, if necessary. High-resolution OCT has been explored to image freshly excised, unstained tissue and provide an earlier feedback^46^,^47^. The high spatial resolution of these bench-top systems offers a detailed view of the various tissue structures, however, scattering contrast between stromal and cancerous tissue is limited. In addition, this high-resolution imaging is slow and incompatible with imaging through miniature probes. Although needle-based PS-OCT imaging features lower spatial resolution, the birefringence signal is sensitive to the arrangement of fibrillar tissue components, such as collagen, on a microscopic scale, and offers contrast that is complementary to the structural intensity signal of conventional OCT. Our imaging results and the excellent match with histology strongly suggests that PS-OCT provides contrast between malignant, cancerous tissue and the surrounding non-cancerous fibrous and fat tissue. PS-OCT, thus, may offer the ability to identify all important tissue types relevant to assess margin involvement during the resection of breast tumour. Importantly, we performed PS-OCT through miniature imaging probes incorporated into hypodermic needles. These imaging needles overcome OCT’s conventional limitation (and indeed that of all of optical microscopy) of imaging only superficial tissue with a planar geometry and offer the possibility to integrate imaging into the surgical workflow. Rather than imaging tissue after its excision, these probes could allow true intraoperative *in situ* imaging to help guide the resection. Crucially, this does not impair histological assessment of the resected tissue for final validation, but should help in reducing the prevalence of involved margins. Future work will increase the limited number of samples imaged to date, and establish a more complete dictionary of the birefringence signatures in normal breast tissue, breast tumour of various types and stages, as well as in desmoplastic stroma. The same contrast mechanism may also help guiding the resection or needle aspiration of other tumours or lymph nodes.

We expect the increased oriented collagen content of connective and desmoplastic tissue, as compared to malignant tissue, to be the main source of the observed birefringence contrast. The birefringence measurement also depends on the orientation of the optic axis of the tissue with respect to the OCT beam. Only the components of the optic axis orthogonal to the probing beam induce any retardation. However, the constantly changing orientation of the probing beam due to the helical scanning makes it highly unlikely to entirely miss the birefringence of tissue rich in collagen. The benefit of birefringence as a useful imaging contrast is also supported by previous work^48^,^49^ which investigated bench-top PS-OCT and cumulative retardation imaging of breast tumour samples to differentiate between healthy, collagen-rich, and cancerous tissue. Birefringence was also demonstrated as a promising contrast to guide bronchoscopic biopsy of lung tumours^50^.

In addition to the integration of the PS-OCT into miniature imaging probes, which is crucial for future clinical translation, we also achieved important improvements applicable to depth-resolved birefringence imaging in general. We refined depth-multiplexed PS-OCT and developed a novel method, complemented with a validity criterion, for the robust reconstruction of the depth-resolved tissue birefringence. It is important to note that PS-OCT only detects fully polarized and coherent light, which is accurately described by the Jones formalism. However, adjacent pixels not only have independent noise realizations, but can also present a deterministic variation of the measured polarization states because they correspond to a different sample location. Adding adjacent Jones matrices coherently is equivalent to using a lower resolution OCT system. It does not remove speckle, but corresponds to a new realization of speckle, with an increased size. This reasoning applies both to the axial and the lateral direction. To overcome this deficit, previous efforts aimed at aligning the global phase of the Jones matrices to ensure their constructive interference^25^,^30^,^31^,^38^. This avoids the creation of signal-void ‘dark’ speckle, but is complicated, remains subject to occasional artefacts, and lacks physical meaning. Averaging, instead, the corresponding Mueller-Jones matrices incoherently is straightforward, reduces speckle contrast, and improves the SNR. This is critical for accurate and robust measurements of depth-resolved birefringence. The Mueller formalism, thus, offers a convenient toolkit to analyse PS-OCT and the deterministic variation of the measured polarization states within the averaging kernel translates to depolarization, which can provide additional contrast^41^. We also have defined, for the first time, a criterion to estimate the validity of the recovered birefringence. Because each sample layer is imaged through the preceding tissue, we are only able to obtain a similarity transformation of each tissue layer. Our criterion estimates the possible error in the extracted birefringence as a function of the polarization properties of the preceding tissue, and can be directly computed from the experimental measurements. The same criterion also limits the Jones formalism, but cannot be specified in the Jones domain, because it ignores depolarization.

The Mueller formalism has previously been used for the analysis of PS-OCT data. In interesting early work, Jiao *et al*. have developed a time-domain PS-OCT system that, within a single depth scan, measured the full Jones matrix. They have then constructed the corresponding Mueller-Jones matrices to analyse biological samples^51^,^52^,^53^. However, without performing spatial averaging, the analysis in the Mueller formalism is equivalent to that in the Jones formalism, and does not offer any specific advantage. Related to the Mueller formalism, Stokes vector analysis has also been used to process PS-OCT data, especially when modulating the incident polarization state between A-lines^22^,^54^. Using input states that are orthogonal to each other on the Poincaré sphere avoids the necessity for phase stability between A-lines, and also benefits from incoherent averaging^37^. It relies, however, on the assumption that the sample acts solely as a retarder, and does not offer the robustness and refinement of the full Mueller formalism.

We have previously shown the detrimental effect of polarization mode dispersion (PMD) on PS-OCT^55^. Circulators, commonly used in fibre-based imaging systems, are a prominent source of PMD and can cause severe birefringence artefacts^37^. We and others have developed strategies to compensate for such system-induced PMD^38^,^56^,^57^,^58^. The PS-OCT system in this study showed negligible PMD, despite the employed fibre circulator, and compensation for PMD was unnecessary. If needed, the concept of ‘spectral binning’^57^ could be applied to the presented formalism.

In summary, the PS-OCT platform introduced here employs miniature imaging probes and a robust reconstruction methodology for imaging of deep tissue layers with birefringence as additional, intrinsic contrast. Pilot imaging of breast cancer samples with this powerful instrument revealed clear birefringence contrast between uninvolved stroma and tumour. Such contrast represents a potential solution to a long-standing problem in OCT imaging of cancer. In the specific case of breast cancer, PS-OCT through a needle could enable improved assessment of tumour margins during the resection of breast cancer.

## Methods

### Needle probes

24G imaging needles with an anastigmatic design were used, as previously reported^8^, and presented in Fig. 1c. Briefly, needles were fabricated by splicing a segment of no-core (i.e., coreless) fibre with a length of ~270 μm (NCF125, POFC, Chu-Nan, Taiwan) to the single-mode fibre (SMF28, Corning Inc., Corning, USA), followed by a segment of graded-index fibre with a length of ~110 μm (GIF625, Thorlabs, Newton, USA), and terminated with a final segment of coreless fibre, which was then angle-polished to deflect the beam at 96° by total internal reflection. This fibre assembly was fused into a collapsed glass capillary to maintain the reflection when embedded in optical adhesive. In a last step, the probe assembly was glued into a hypodermic needle and aligned with a side window drilled by electrical discharge machining. The needles featured a 1/e^2^ spot diameter of ~20 μm (corresponding to a full-width at half-maximum lateral resolution of ~12 μm) in a focal plane located ~300 μm from the needle surface^8^, and with a depth of focus of ~650 μm in tissue with a refractive index of n = 1.36 (for reasons explained in ref. 8, the focal spot of this design was slightly elliptical with measured 1/e^2^ diameters of 22 μm and 18 μm in the directions parallel and orthogonal to the needle axis, respectively. The given resolution and DOF, therefore, represent an average of the two axes).

### PS-OCT system

The OCT system was driven by a wavelength-swept laser source (AXP50125-6, Axsun, Billerica, USA), centred at 1310 nm, with a full sweep bandwidth of 100 nm and sweep rate of 50 kHz. In the sample arm, two orthogonal polarization states were passively multiplexed in depth by introducing a differential path length (~3.8 mm), as in ref. 23. A polarization-diverse balanced receiver was implemented with a polarization-diverse optical mixer (PDOM-1310, Finisar, Sunnyvale, USA), the four optical outputs of which were connected to two identical balanced detectors (PDB460C-AC, Thorlabs, Newton, USA). Their output signals were low-pass filtered with a cutoff frequency of 120 MHz before analogue-to-digital conversion using a dual-channel digitizer card (ATS9350, Alazar, Pointe-Claire, Canada), which was clocked with the frequency-doubled “k-clock” signal of the laser source, similar to ref. 25. The frequency doubling extended the available depth range to accommodate the depth-multiplexed polarization states. The sensitivity was measured to be >110 dB in a single detection channel when using a single input state and optimizing the output signal in that detection channel. The power incident on the sample was 8.2 mW for each input polarization state. The 6 dB intensity signal roll-off was measured to be 7.8 mm, and was calibrated using a single-input polarization state and displacing a sample mirror along the entire imaging range. The roll-off was fitted with a 2^nd^ order polynomial, which was used to correct the raw tomograms to ensure equal signal amplitude between the two depth-multiplexed input polarization states. The tomogram of each detection channel was then split at the differential path-length offset to retrieve the depth-multiplexed input polarization states. Knowing the precise offset is critical, and it was determined by analysing the auto-correlation along depth of the first B-scan, whenever the delay had been altered.

For system validation and imaging of fish tissue, the needle probes were mounted on a rotation/pullback setup comprising a fibre-optic rotary joint (MJP-FAPB-131-28-FA, Princetel, Hamilton, USA,) driven by a DC motor (EC22, Maxon, Sachseln, Switzerland) and mounted on a motorized translation stage (GTS70, Newport, Irvine, USA). This setup performed helical scanning at 2 rotations/s with a pullback speed of 20 μm/s and an angular sampling density of 1800 A-lines per 360°, achieved by down-sampling the original A-line rate. For imaging of the tumour specimens in a hospital setting, the probes were attached to a more compact and lightweight custom-built motorized rotation/pullback assembly which could be mounted on an articulating arm for insertion of the needles into excised lumps of tissue at arbitrary positions and angles. This assembly did not incorporate a rotary joint in order to save weight and, therefore, scanned a cylindrical volume by counter-rotating back and forth over 360 degrees at 1 Hz and pulling back in 13.2 μm steps between counter-rotations, with an angular sampling density of 1600 A-lines per 360°.

### Phase jitter removal

The optical clocking of the acquisition assured that sampling was linear in the wavenumber *k*. However, there remained a timing jitter caused by an inherent uncertainty in the precise *k*-value of the very first trigger signal of each A-line. A jitter of a single clock cycle results in a relative linear phase running from 0 to 2π across the depth range. Because of the depth encoding, this creates a constant phase offset between the two columns of the Jones matrix. Although the Mueller processing is insensitive to the absolute phase of the recovered Jones matrix, this phase offset alters the reconstructed Mueller matrix, and would impair averaging of Mueller matrices across A-lines if left uncorrected. To identify and correct the A-lines that experienced an offset trigger signal, we computed the intensity-weighted mean phase difference between A-lines of the relative phase of the input polarization states:


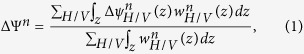


where *H* and *V* are the two detected polarization states, and


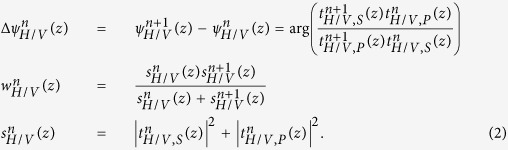


Here, *n* is the A-line index, *t*_*H/V,S/P*_ the tomogram of detection channel *H* and *V*, and input states *S* and *P*, respectively. Without timing jitter, ΔΨ^ n^ is centred at zero. However, in about 50% of A-lines, a premature or delayed triggering created a phase offset of ±2π*z*_*PDU*_/*z*_*tot*_, where *z*_*tot*_ is the entire available ranging depth, and *z*_*PDU*_ is the path-length offset between the depth-encoded input polarizations. Identifying these instances with a threshold and correcting for the *a priori* known phase offset was straightforward, and led to consistent Jones and, eventually, Mueller matrices.

### Reconstruction of tissue birefringence

The combination of the two detection channels and the two input polarization states provides directly the round-trip Jones matrix at each point in the tomogram:


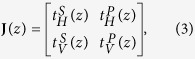


where *t*_*H/V*_^*S/P*^ is the complex-valued tomogram of detection channel *H, V* and input polarization state *S, P*, after roll-off correction and phase-jitter removal. This Jones matrix **J**(*z*) can be directly transformed into the corresponding Mueller-Jones matrix:


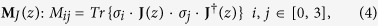


where *Tr* indicates the trace of a matrix, *σ*_*i*_ are the Pauli matrices, and the dagger denotes the complex transpose. Each element of the calculated Mueller matrix was filtered with a Gaussian kernel, extending by *w*_x_ = 2° and *w*_z_ = 32 μm along the rotational and the axial directions, respectively. This kernel size corresponds to about two axial speckle widths and slightly more than two lateral speckle widths, effectively averaging an area encompassing about five speckles.

For the following, it is important to take into account that the Mueller matrix corresponds to measurements taken in reflection mode. Accordingly,


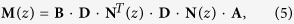


where **A** and **B** describe the transmission through the imaging system from the polarization delay unit to the sample surface, and from there to the receiver, respectively, and are generally well described as pure retarders. **N**(*z*) is the single-pass transmission matrix from the sample surface to the depth *z*, and **D**⋅**N**^T^(*z*)⋅**D** defines the reverse, passing through the sample from depth *z* to the sample surface, where **D** = diag(1, 1, 1, −1) is a diagonal matrix. This is equivalent to taking the matrix transpose in the Jones formalism.

To extract tissue birefringence at a given depth, we have to compute the retardation over a differential depth ∆*z*:


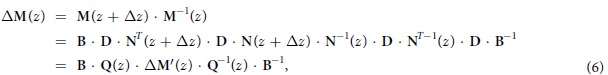


where


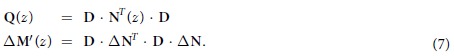


Here, Δ**N** is the single-pass matrix from *z* to *z* + ∆*z*, Δ**M′** the double pass matrix from *z* to *z* + ∆*z* and back to *z*, and Δ**M** is the similarity transformation of Δ**M′**, defined analogously to the Jones formalism^28^. ∆*z* was set to compute Δ**M** between directly adjacent depth pixels. To accelerate the reconstruction and avoid the computationally intensive matrix inversion, we approximated **M**^−1^ with a pseudo-inverse that is more efficient to compute:


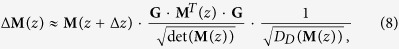


where **G** = diag(1, −1, −1, −1) is the Minkowski matrix and *D*_*D*_ the depolarization index:


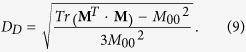


The pseudo-inverse exactly corresponds to the true inverse matrix when **M** is a non-depolarizing Mueller matrix. Accurate retardation is also recovered (by division by the square root of the depolarization index) if the canonical decomposition of **M**(*z*) features retardation and/or isotropic depolarization. As discussed and analysed, additional diattenuation and anisotropic depolarization limit the accuracy of the recovered retardation.

To retrieve the retardation, we next approximated the matrix logarithm **m**(*z*) = logm(∆**M**(*z*)) ≈ ∆**M**(*z*)/det(∆**M**(*z*))^1/4^ − **I**, where **I** is the identity matrix, to obtain an estimation of the differential Mueller matrix^35^. From there, we extracted the local retardation *γ* by computing the **G**-symmetric differential matrix and taking the norm of the retardation vector, averaged over an axial distance *w*_*z*_, matching the size of the spatial filter originally used to generate **M**(*z*):


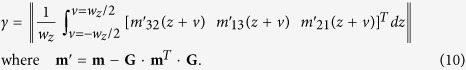


This corresponds closely to the retardation of ∆**M**(*z*) = **M**(*z* + *w*_*z*_/2)⋅**M**^−1^(*z* − *w*_*z*_/2), but minimizes the error introduced by the approximation of the matrix logarithm. Dividing by twice the differential depth ∆*z* and the central wavenumber *k*_c_, we then obtained the depth-resolved tissue birefringence ∆*n = γ*/(*k*_*c*_2∆z).

In the current, un-optimized Matlab implementation, reconstruction of a birefringence image, with 400 depth pixels and 1600 A-lines, takes 2.5 seconds. The implementation with the accurate matrix inverse takes more than 2 minutes.

### Imaging tumour specimens

Imaging was performed at Sir Charles Gairdner Hospital in Perth, Western Australia. Informed consent was obtained from the patients and the study was approved by the Human Research Ethics Committee of Sir Charles Gairdner Hospital, Perth, Western Australia, and carried out in accordance with the approved protocol. For the current preliminary study, two tissue samples were imaged from patients undergoing a lumpectomy and mastectomy, respectively. After imaging with needle-based PS-OCT, the tissue was fixed in 10% neutral-buffered formalin for 24 hours, then processed, sectioned and stained with haematoxylin and eosin (H&E) following the standard protocol used at Sir Charles Gairdner Hospital. H&E stained sections were digitally micrographed (ScanScope, Leica Biosystems) and manually co-registered with the PS-OCT scans using in-house viewing software.

## Additional Information

**How to cite this article**: Villiger, M. *et al*. Deep tissue volume imaging of birefringence through fibre-optic needle probes for the delineation of breast tumour. *Sci. Rep.* **6**, 28771; doi: 10.1038/srep28771 (2016).
